# Identification of novel microRNAs in the sheep heart and their regulation in heart failure

**DOI:** 10.1038/s41598-017-08574-x

**Published:** 2017-08-15

**Authors:** Lee Lee Wong, Miriam T. Rademaker, Eng Leng Saw, Kar Sheng Lew, Leigh J. Ellmers, Christopher J. Charles, Arthur Mark Richards, Peipei Wang

**Affiliations:** 1 Cardiovascular Research Institute, Yong Loo Lin School of Medicine, National University Health System, National University of Singapore, Singapore, Singapore; 2 Department of Medicine, Centre for Translational Medicine, Yong Loo Lin School of Medicine, National University Health System, National University of Singapore, Singapore, Singapore; 3 Christchurch Heart Institute, Department of Medicine, University of Otago-Christchurch, Christchurch, New Zealand; 4 Cardiac Department, National University Health System, Singapore, Singapore; 5 Department of Surgery, National University of Singapore, Singapore, Singapore

## Abstract

Study of microRNA (miRNAs) using sheep models is limited due to lack of miRNA information. We therefore investigated oar-miRNAs and their regulation in an ovine model of heart failure (HF). Left ventricular (LV) tissue was collected from normal (Cont), HF (LV pacing @ ~220bpm for 13-days) and HF-recovery sheep (HF-R, 26-days after pacing cessation). MiRNA expression was profiled using next-generation sequencing (NGS) and miRNA array, and validated by stem-loop qPCR. Detected sequences were mapped against the ovine genome (Oar v4.0) and aligned with known miRNAs (miRBase v21). A total of 36,438,340 raw reads were obtained with a peak distribution of 18–23 nt. Of these, 637 miRNAs were detected by NGS and mapped to the ovine genome. With cut-off at 10 counts, 275 novel miRNAs were identified (with 186 showing 100% alignment and 89 showing 70–99% alignment with human/mouse and/or rat miRNAs, respectively), and 78 known oar-miRNAs. Cardiac-enriched miRNA-1, -133a, -208a/b and -499 were highly expressed in the LV. With HF induction, miRNA-133b-3p, -208b-3p, -125a-5p, -125b-5p, -126-3p, -21-5p, -210-3p, -29a-3p, -320a and -494-3p were significantly up-regulated relative to Cont and tended to return to normal levels following HF-recovery. This study has expanded the sheep miRNA database, and demonstrated HF-induced regulation of miRNAs.

## Introduction

Since the first discovery of microRNA (miRNA) lin-4 in 1993^1^, it has become clear that miRNAs play key roles in regulating both normal development and the response to injury in cardiovascular health and disease. They are also potential candidates as diagnostic biomarkers and therapeutic targets in cardiovascular disease including heart failure (HF)^2, 3^.

To date, pre-clinical investigations into the importance of miRNA in cardiovascular disease have been predominantly limited to small animal models and cell-based studies. Large animal models, however, are particularly valuable in translational research due to size, anatomy, physiology and genetic similarities to human beings^4^. Sheep, with a similar body/organ size to humans and comparable coronary artery configuration, in addition to their docile behavior and resilience in regard to surgery and infection, provide an extremely useful animal model for research in cardiovascular surgery and devices, genetic disorders and investigations into the pathophysiology of HF^4–6^. The ovine rapid ventricular pacing model of HF, in particular, has been utilized extensively in the study of biological pathways involved in HF, especially those relating to neurohormonal systems, and in the development and refinement of HF therapies^7–9^. This model closely replicates the hemodynamic, endocrine and metabolic characteristics of severe low-cardiac output HF in humans^10^, with the generation of cardiac and kidney dysfunction and activation of neurohormonal systems occurring in a time-dependent and highly predictable fashion.

Lack of information on sheep miRNAs in existing databases has limited the use of ovine models in cardiovascular research. In the latest miRBase v21 database (http://www.mirbase.org/), there are a total of 28,645 miRNA entries from various species, of which 2585 and 1870 correspond to mature and hairpin precursor human (hsa-) miRNA, 1899 and 1180 to mouse (mmu-) miRNA, and 788 and 484 to rat (rno-) miRNA. However, there are only 154 mature and 109 hairpin precursor miRNAs described for sheep (oar-), making this a particularly underexplored field in this species. In addition, most oar-miRNAs have been identified from ovary^11, 12^, skeletal muscle^13^, liver^14^, mammary gland^15^ and wool and hair follicles^16^. To date, there have been few miRNA-related studies performed in sheep cardiac tissue^17^, and none in the setting of HF.

A number of different methods are used in the identification of miRNAs, including stem-loop qPCR which is considered a gold standard for miRNA validation, and miRNA array profiling which is a highly sensitive platform that has allowed identification of most known hsa-/mmu-/rno-miRNAs^18^. In recent years, next-generation deep sequencing (NGS) technology has also become available that allows detection of novel transcripts including miRNAs^19^. It is possible that the simultaneous performance of all three methods may improve the ability to identify novel miRNAs on a large scale. Therefore, we attempted to expand the oar-miRNA database by examining sheep left ventricular (LV) tissue using NGS, miRNA array and qPCR techniques, and to investigate cardiac miRNA regulation in the well-established sheep rapid-pacing model of HF.

## Results

### Hemodynamic and neurohumoral changes in ovine HF and HF-R

Thirteen days of rapid LV pacing produced the hemodynamic and neurohumoral hallmarks of established HF, with marked decreases in cardiac output (CO) and mean blood pressure (MBP), and rises in atrial pressure and plasma atrial and B-type natriuertic peptides (ANP and BNP, resepectively) (all p < 0.001 vs. Cont). These changes returned to baseline levels following 26 days recovery from HF, with the exception of still slightly reduced MBP and elevated plasma ANP (both p < 0.05 vs. Cont). Data are summarized in Table 1.

**Table 1:** Hemodynamic and neurohormonal changes in an ovine model of rapid pacing-induced heart failure and recovery.

	Control (n = 13)	HF (n = 6)	HF-R (n = 8)
**Hemodynamic Measurements**			
Heart Rate (bpm)	80 ± 4	218 ± 2***	84 ± 5^†††^
Cardiac Output (L/min)	7.13 ± 0.22	2.93 ± 0.13***	7.11 ± 0.29^†††^
Mean Blood Pressure (mm Hg)	101.0 ± 2.2	71.1 ± 2.8***	87.5 ± 3.9*^††^
Atrial Pressure (mm Hg)	5.00 ± 0.71	28.0 ± 0.88***	5.88 ± 0.50^†††^
**Neurohumoral Measurements**			
ANP (pmol/L)	16.1 ± 3.9	297.3 ± 26.5***	23.9 ± 2.0*^†††^
BNP (pmol/L)	2.85 ± 0.75	55.3 ± 9.33***	2.6 ± 0.46^††^

Mean ± SEM measurements in normal Control sheep and sheep subject to heart failure induced by rapid left ventricular pacing at ~220 bpm for 13 days (HF) and following recovery from heart failure after 26 days cessation of pacing (HF-R). Atrial natriuretic peptide (ANP); B-type natriuretic peptide (BNP). *P < 0.05 and ***P < 0.001 vs Control; ^††^ *P* < 0.01 and ^†††^ *P* < 0.001 vs HF.

### Overview of next generation deep sequencing miRNA data

**Figure 1:**
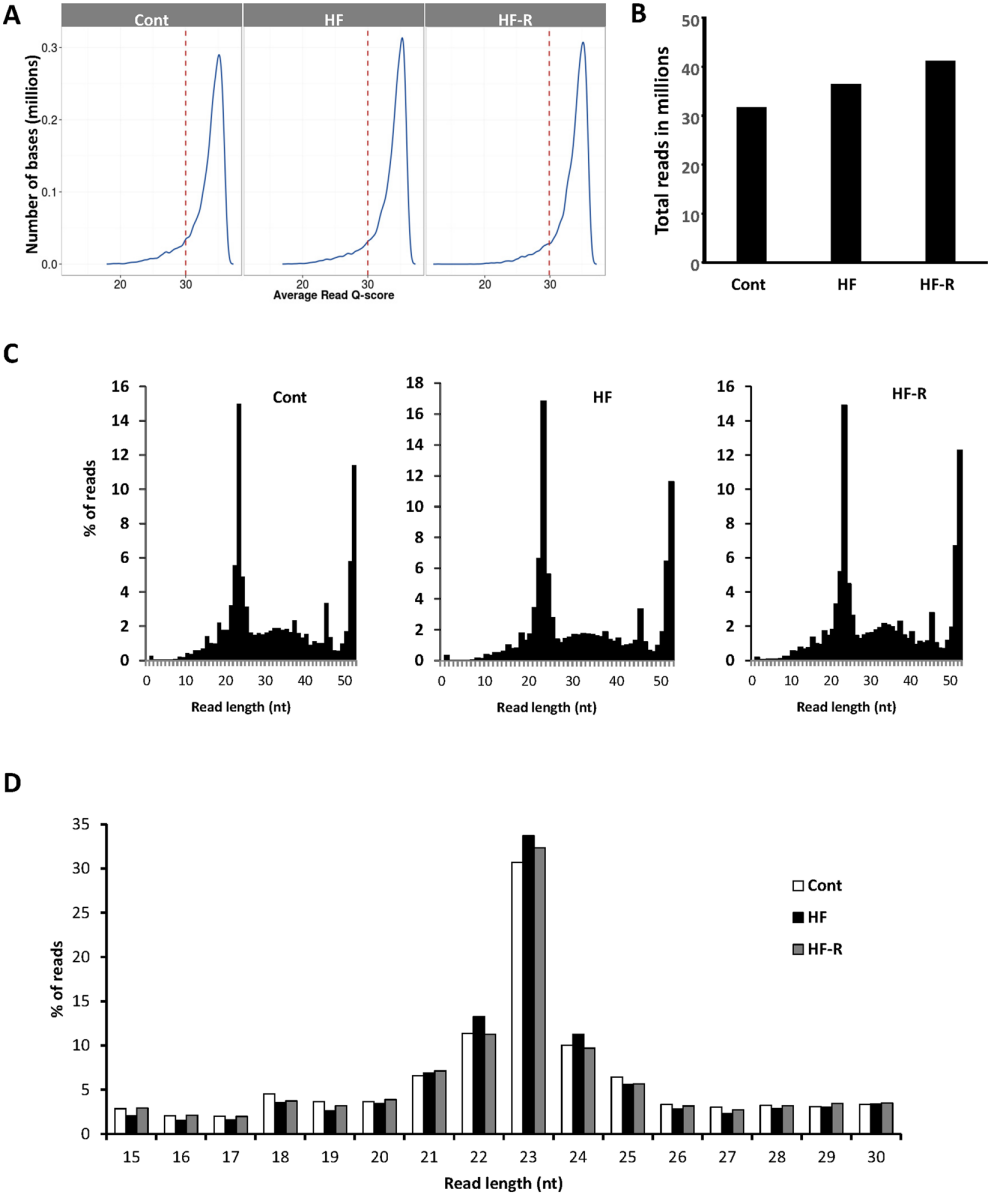
Overview of next generation deep sequencing (NGS) reads analysis. (**A**) The average Q-scores of the NGS sequencing data. (**B**) Total number of reads per group. (**C**) Overall nucleotide (nt) distribution. (**D**) Distribution of sequences between 15–30 nts representing miRNAs. Samples are pooled sheep left ventricular (LV) tissue from normal Controls (Cont, n = 4), Heart Failure (HF, n = 6) and Heart Failure-Recovery (HF-R, n = 4).

**Figure 2:**
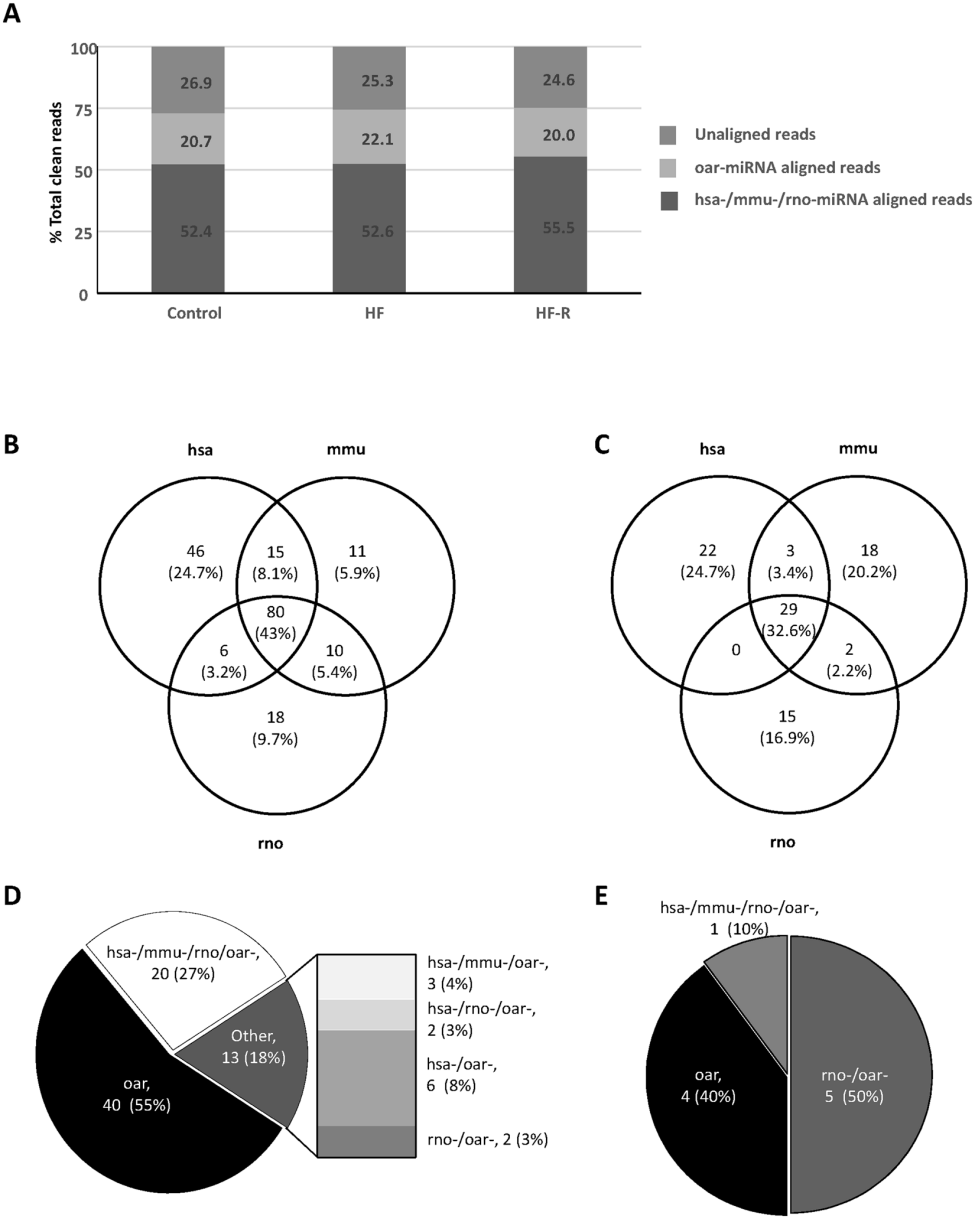
Analysis of NGS miRNA sequences. (**A**) Summary of NGS reads of 15–30 nts alignment to hsa-, mmu- and rno- and known oar-miRNAs (miRBase v21 miRNA). (**B** and **C**) Distribution of 186 novel oar-miRNAs perfectly aligned (100%) and 89 oar-miRNAs 70–99% aligned, respectively, with hsa-, mmu- and rno- miRNAs. (**D** and **E**) Distribution of 73 known oar-miRNAs aligned perfectly and 10 aligned 70–99% with hsa-, mmu- and rno- miRNAs.

The Q-scores were higher than 30 for all three libraries. After trimming off the sequence adapters from the dataset, the libraries averaged a total of 36.44 million raw reads. Sequences were distributed into two peaks at the lengths of ∼40–50 and ∼15–30 nts which represent RNA fragments and miRNAs, respectively. Close examination of the small RNA sequences revealed that sequences of 22–25 nts accounted for ~80% of the total sequence counts of 15–30 nts (Fig. 1). The 17.69 million clean counts sequences of ∼15–30 nts were mapped to the sheep genome (Oar v4.0). They were further classified into three categories (Fig. 2A): (1) Sequences of 53.5% counts which were novel oar-miRNAs aligning with hsa-, mmu- and rno-miRNAs. (2) Sequences of 20.9% counts which were known oar-miRNAs aligning with oar-miRNAs. (3) Sequences of 25.6% counts which were not aligned with any known miRNAs. These sequences could be putative miRNAs and require further validation.

An in-depth analysis was performed to identify novel sheep miRNAs. A total of 637 miRNAs were identified by NGS in sheep LV tissue. Of this total, 535 miRNA had not previously been reported in sheep and 102 were known oar-miRNAs, of which 275 and 83 miRNAs, respectively, demonstrated counts ≥10. Of the 275 unidentified miRNA, 186 aligned 100% with one or more hsa-, mmu- and rno-miRNAs (Fig. 2B, Supplementary Table S1). These are most certainly authentic novel oar-miRNAs. The remaining 89 miRNA shared 70–99% homology with one or more hsa-, mmu- and rno-miRNAs (Fig. 2C, Supplementary Table S2). These are also likely to be novel oar miRNAs. Similarly, of the 83 known oar-miRNA sequences, 73 miRNAs were perfectly aligned with oar-miRNAs (Fig. 2D, Supplementary Table S3) and 10 at least 70% aligned (Fig. 2E, Supplementary Table S4). Forty four miRNAs were unique oar-miRNAs, while the remaining oar-miRNAs were conserved with one or more hsa-, mmu-, rno-miRNAs.

### Overview of miRNA array data

**Figure 3:**
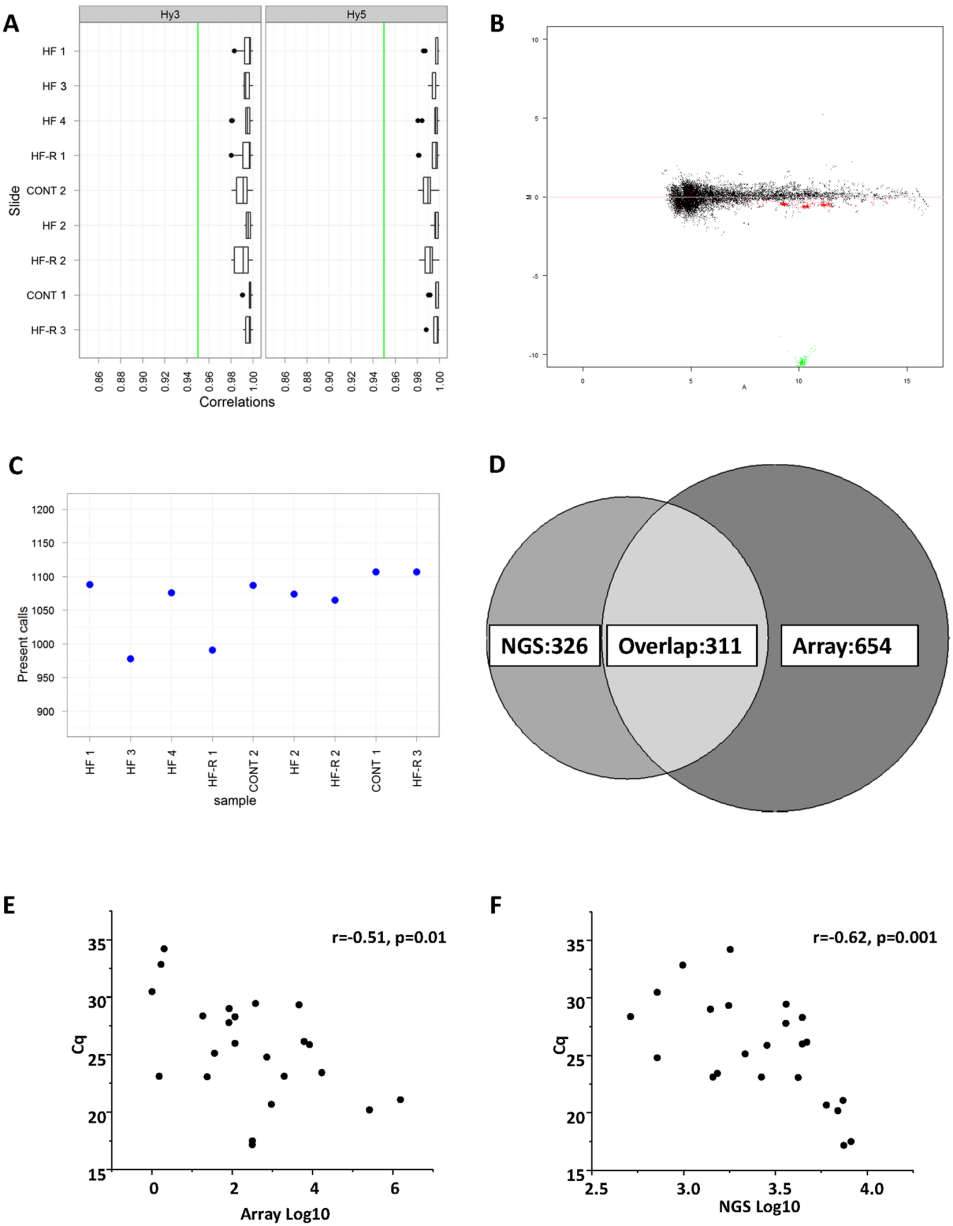
Analysis of microRNA microarray data from sheep left ventricular tissue. (**A**) Box plot showing the correlation R^2^ of 52 spike-ins for each sample with Cy3 and Cy5 labelling. Black dots indicate outliers. (**B**) MA-plot of a representative sample after Lowess (LOcally WEighted Scatterplot Smoothing) normalization. Green: Hy3 controls, red: spike-ins, black: all other probes. (**C**) Number of miRNA in each individual sample detected above background threshold (1.2 fold above 25^th^ percentile). (**D**) The overlap of miRNAs detected by next generation deep sequencing (NGS) and miRNA array. (**E** and **F**) Correlation of array intensities with NGS counts and qPCR Cq values for 23 miRNAs. (Cont n = 2, HF n = 4, HF-R n = 3).

To verify NGS miRNA findings, an independent miRNA array was performed in 9 individual samples (2 Cont; 4 HF; 3 HF-R). Fifty two spike-in controls covering the full signal intensity range in both the Hy3™ and the Hy5™ labelling reactions were added to ensure array quality assessment. The correlation (R^2^) of all spike-ins across all samples were ~0.99 (Fig. 3A). Locally weighted scatterplot smoothing (Lowess) normalization, performed to minimize systematic variations, are illustrated in MA-plots (M: log2 Cy5−log2 Cy3; A: mean of log2 Cy5 + log2 Cy3. After normalization, the dots scattered symmetrically around the horizontal line M = 0 and miRNA abundance was dependent on the average intensity of Cy3 and Cy5 (Fig. 3B one out of nine representative data). The normalized data were then subjected to unsupervised hierarchical clustering analysis. By setting the background threshold cut-off at 1.2 times the 25^th^ percentile (range 5.1–16.2) of the overall signal intensity of each slide, 965 miRNAs were detected in at least two samples (Fig. 3C, Supplementary Table S5). By a comparison of these miRNAs with 637 miRNAs identified by NGS, we found an overlap of 311 miRNAs (Fig. 3D). To further verify the miRNAs detected by NGS and microarray, randomly selected 23 miRNAs with miRNA microarrays signal intensities ranging from lowest to highest were measured by qPCR. qPCR validation showed Cq values were negatively correlated with both array (r = −0.51; Fig. 3E) and NGS (r = −0.62, Fig. 3F).

### Identification of cardiac-enriched miRNAs

MiRNA-1, -133a, -208a/b and -499 are reported as cardiac-specific or cardiac-enriched miRNAs in many species including the mouse, rat and human^20^. Of these, oar-miRNA-133 is the only one presently recorded in miRBase (v21). In the current study, NGS detected all 4 of these miRNAs in sheep LV tissue. They were conserved with one or all hsa-, mmu- and/or rno-miRNAs and were highly expressed (Table 2). Hsa-/mmu-miR-1a-3p, which has the same sequence as rno-miR-1b, showed high expression levels in the sheep heart. Mmu-miR-1b-5p should be the passenger strand of this miRNA. Oar-miR-133 was the main form in sheep heart, while hsa-/mmu-/rno-miR-133a-3p and -5p and hsa-/mmu-/rno-miR-133b were detected at much lower counts. Hsa-/mmu-/rno-miR-208b-3p was the most abundant form of the miR-208 family. However, the expression level was low compared to the other cardiac-enriched miRNAs. Hsa-/mmu-/rno-miR-499a-5p and hsa-499b-3p were plentiful in sheep heart whereas the expression of other forms of miR-499 were low.

**Table 2:** Identification of cardiac-specific microRNAs in the sheep heart.

miRNA_ID	Chr_ID	Strand	Start	End	Sequence	Counts* 100%	Counts* 70–99%	Array	Cq
hsa-/mmu-miR-1a-3p, rno-miR-1b	NC_019480.2	+	34708115	34708136	TGGAATGTAAAGAAGTATGTAT	1525891	566849	14.7	21.8
	NC_019470.2	−	53971898	53971919					
mmu-miR-1a-2-5p	NC_019480.2	+	34708076	34708098	ACATACTTCTTTATGTACCCATA	1	4472	9.9	
mmu-miR-1b-5p	NC_019470.2	+	53971899	53971919	TACATACTTCTTTACATTCCA	54503	557416	6.1	
	NC_019480.2	−	34708115	34708135					
oar-miR-133 (-3p)	NC_019470.2	−	53961766	53961787	TTGGTCCCCTTCAACCAGCTGT	54412	145615		
hsa-/mmu-/rno-miR-133a-3p	NC_019480.2	+	34711428	34711449	TTTGGTCCCCTTCAACCAGCTG		312	14.7	17.2
	NC_019470.2	−	53961767	53961788					
hsa-/rno-miR-133a-5p	NC_019480.2	+	34711391	34711412	AGCTGGTAAAATGGAACCAAAT	1893	1258	11.1	
	NC_019470.2	−	53961804	53961825					
mmu-miR-133a-5p	NC_019480.2	+	34711392	34711412	GCTGGTAAAATGGAACCAAAT	25	1241		17.5
	NC_019470.2	−	53961804	53961824					
hsa-/mmu-/rno-miR-133b	NC_019477.2	+	24293671	24293692	TTTGGTCCCCTTCAACCAGCTA		315	15.1	
mmu-miR-133b-5p	NC_019477.2	+	24293634	24293655	GCTGGTCAAACGGAACCAAGTC		11		
hsa-/mmu-miR-208a-3p	NC_019464.2	+	21140684	21140705	ATAAGACGAGCAAAAAGCTTGT	41	122	6.7	
rno-miR-208a-3p	NC_019464.2	+	21140684	21140701	ATAAGACGAGCAAAAAGC	16	108	6.7	
hsa-/mmu-/rno-miR-208a-5p	NC_019464.2	+	21140649	21140670	GAGCTTTTGGCCCGGGTTATAC	1		7.3	
hsa-/mmu-miR-208b-3p	NC_019464.2	+	21111702	21111723	ATAAGACGAACAAAAGGTTTGT	6099	7710	12.8	26.2
	NW_014640758.1	−	2651	2672					
mmu-miR-208b-5p	NC_019464.2	+	21111667	21111688	AAGCTTTTTGCTCGCGTTATGT	17	39	7.3	
	NW_014640758.1	−	2686	2707					
hsa-/rno-miR-499a-3p	NC_019470.2	+	63735196	63735217	AACATCACAGCAAGTCTGTGCT	375	1178	11.8	29.5
mmu-miR-499-3p	NC_019470.2	+	63735195	63735217	GAACATCACAGCAAGTCTGTGCT	11	1180		
hsa-/mmu-/rno-miR-499a-5p	NC_019470.2	+	63735159	63735179	TTAAGACTTGCAGTGATGTTT	269451	160221	14.3	20.2
hsa-miR-499b-3p	NC_019470.2	−	63735157	63735178	AACATCACTGCAAGTCTTAACA	259451	159579		
hsa-miR-499b-5p	NC_019470.2	−	63735194	63735214	ACAGACTTGCTGTGATGTTCA	13	1171	9.1	

*Mean counts of NGS sequencing. The detected sequences were 100% or 70–99% matched to existing miRNA sequences.

### miRNA dysregulation in HF and HF-R using miRNA array profiling and Stem-loop qPCR validation

**Figure 4:**
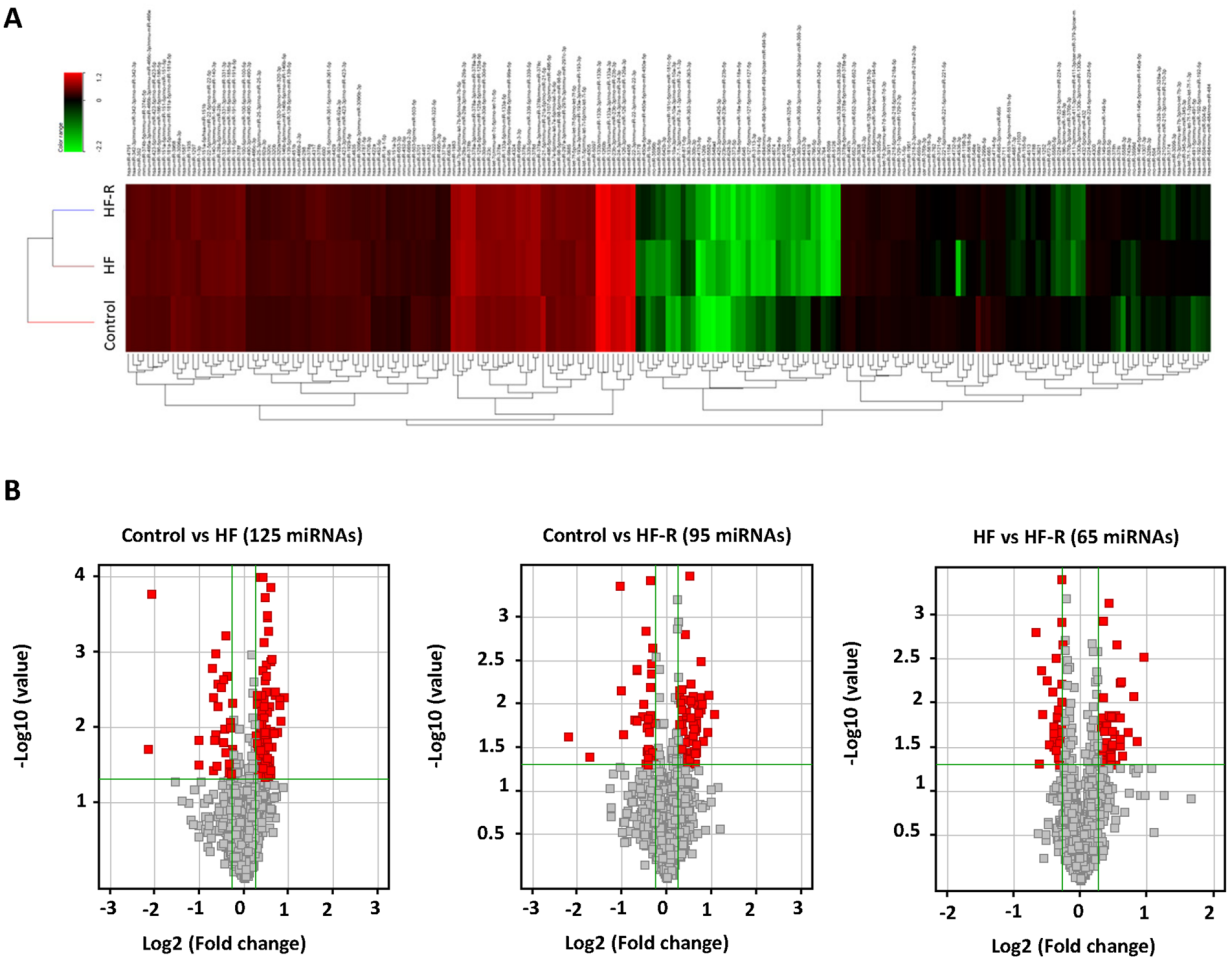
Profiling of miRNA expression. (**A**) Hierachical clustering of miRNA expression (**B**) Volcano plots showing differentially expressed miRNAs with fold change > ±1.2 Cont vs HF, Cont vs. HF-R, and HF vs HF-R (p < 0.05).

Hierarchical clustering of miRNA profiles showed differential miRNA expression between the Control, HF and HF-R groups, with HF and HF-R more different from Cont (Fig. 4A). In total, 125, 95 and 65 differentially expressed miRNAs were identified between Cont vs. HF, Cont vs. HF-R and HF vs. HF-R, respectively, with fold changes >1.2, p < 0.05 (Fig. 4B and Supplementary Table S6).

**Figure 5:**
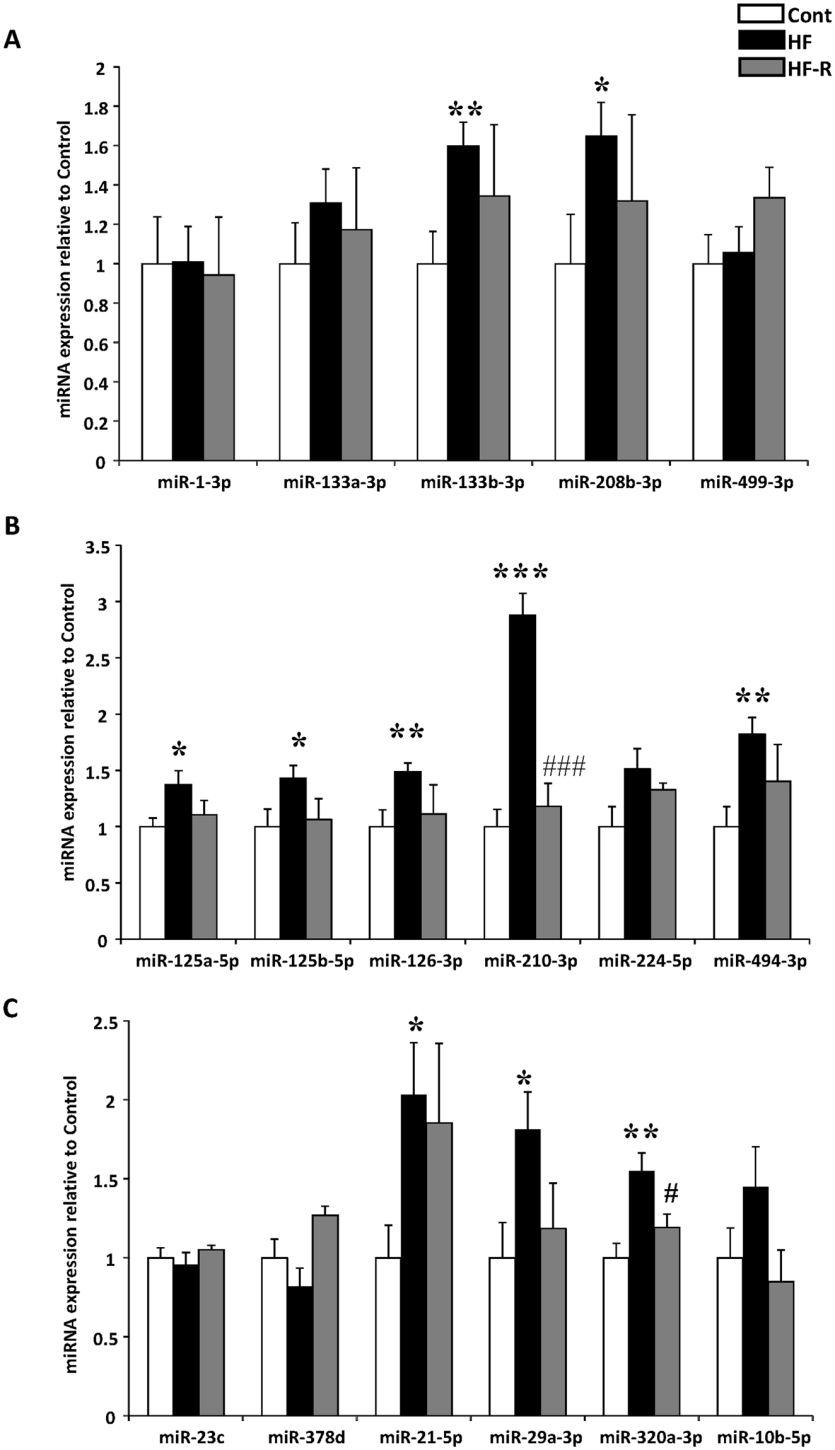
Stem-loop qPCR validation of miRNAs dysregulation. (**A**) Comparison of five cardiac enriched miRNAs in the 3 groups. (**B** and **C**) validation of 12 significantly dysregulated miRNAs detected by miRNA array. *p < 0.05, **p < 0.01, ***p < 0.001 compared with Control; ^#^p < 0.05, ^###^p < 0.001 compared with HF (Student’s t-test). Control (Cont, n = 13), Heart Failure (HF, n = 6) and Heart Failure-Recovery (HF-R, n = 8).

HF-induced miRNA changes were further validated by stem-loop-qPCR. Cardiac-enriched miR-1-3p, miR-133a-3p, miR-133b-3p, miR-208b-3p and miR-499-3p were screened. Of these, miR-208b-3p and miR-133b-were significantly up-regulated in HF and returned towards normal levels in HF-R (Fig. 5A). As a proof of concept study, 12 miRNAs showing significant differences in array (HF vs. Cont or HF-R) were validated by qPCR. Eight miRNAs, namely miR-125a-5p, -125b-5p, -126-3p, -210-3p, -494-3p, -21-5p, -29a-3p and -320a were significantly up-regulated in HF (vs. Cont) and tended to return to Cont levels in HF-R (Fig. 5B and C). The comparison of qPCR, NGS and array data on these miRNAs are summarized in Table 3.

**Table 3:** Summary of HF-regulated microRNAs detected by next generation deep sequencing (NGS), differential expression by miRNA array and validation by stem-loop qPCR.

Annotation	Mean Counts	Mean Intensity	Fold change	Fold change
	NGS	Array	Array	RT-qPCR
hsa-/mmu-miR-1-3p/rno-miR-1b	1525891	14.7		1.01
hsa-/mmu-/rno-miR-133a-3p	312	14.7		1.31
hsa-/mmu-/rno-miR-133b-3p	315	15.1		1.60**
hsa-/mmu-/rno-miR-208b-3p	6099	12.8		1.65*
hsa-/mmu-/rno-miR-499-3p	375	11.8	1.43*	1.06
hsa-/mmu-/rno-miR-125a-5p	8302	11.0	1.62*	1.37*
hsa-/mmu-/rno-/oar-miR-125b-5p	933	13.8	1.62*	1.43*
hsa-/mmu-/rno-miR-126-3p	Not detected	13.9	1.50*	1.49**
hsa-/mmu-/rno-miR-210-3p	18	6.7	1.43*	2.88***
hss-/mmu-/rno-miR-224-5p	718	7.3	1.85*	1.52
hsa-mmu-/rno-/oar-494-3p	229	5.7	−1.62*	1.82**
hsa-miR-23c	Not detected	11.0	1.59*	−1.05
hsa-miR-378d	82	11.7	1.53*	−1.22
hsa/mmu/rno-21-5p	2	11.0	−4.23*	2.03*
hsa-/mmu-/rno-/oar-miR-29a-3p	24	12.4	1.77*	1.81*
hsa-/mmu-/rno-320a	1950	8.9		1.55**
hsa-/mmu-/rno-miR-10b-5p	1	7.3		1.45

*P < 0.05, **P < 0.01, ***P < 0.001 versus normal Controls.

## Discussion

The aims of this study were to identify novel sheep miRNA and to explore HF associated miRNA dysregulation. A total of 637 miRNAs sequences were detected by NGS and 358 of them with counts ≥10. Among them, 186 are novel sheep miRNAs with perfect aligning with hsa-, mmu- and/or rno-miRNAs, and 73 are previously known oar-miRNAs (miRBase v21). These findings were confirmed with independent miRNA array measurements and validation by qPCR analysis for a selected panel of miRNAs. Four myocardial-enriched miRNAs, miR-1, miR-133, miR-499 and miR-208, were confirmed to be highly expressed in ovine heart tissue. Investigation of cardiac miRNA differentially expressed in HF and HF-R by miRNA array analysis followed by qPCR validation identified 10 miRNAs significantly up-regulated in HF, namely miR-133b-3p, -208b-3p, -125a-5p, -125b-5p, -126-3p, -21-5p, -210-3p, -29a-3p, -320a and -494-3p, all of which returned towards normal levels following HF-R.

NGS is a powerful technique in the discovery of novel miRNAs. The critical steps include library preparation, base-calling and read analysis. Since NGS was performed to identify novel oar-miRNAs, pooled samples of each group (Control, HF, HF-R) were used to reduce cost and minimize biological biases from single sample preparation. With all three libraries constructed, the Q-scores were >30, indicating an accuracy above 99.9%^21^. A total of 50 million read depth was used in this study. After trimming off adaptors, 36.44 million reads of sequences were acquired and distributed into two peaks of different length nts. The peak of ∼40–50 length nts representing fragments of rRNA, tRNA and mRNA, while the peak of ∼15–30 length nts representing miRNA sequences with 17.69 million counts. The total miRNA counts were almost doubled compared to the previous studies analyzing miRNAs in sheep^17, 22^. The increase of depth is directly associated with higher accuracy^23^. Therefore more novel miRNA were identified. Overall, 637 miRNA sequences were detected by NGS. All of these mapped to the sheep genome. Taking a conservative criteria to consider the sequences with at least 10 read counts and perfectly aligned with hsa-/mmu-/rno-miRNAs, 186 novel miRNA were identified. In addition, 73 previously known oar-miRNAs sequences with >10 counts were detected. Of the novel and known groups, 89 and 10 miRNA sequences, respectively, demonstrated 70–99% alignment with hsa-/mmu-/rno-miRNAs. Our findings indicate that the 186 perfectly aligned miRNAs are genuine novel oar-miRNAs and the 89 demonstrating 70–99% alignment are also likely to be so. For those sequences not aligned with any known miRNAs, we considered them as putative miRNAs. Further validation is required.

Commercially available miRNA array kits for human, mouse and rat miRNA were used for profiling miRNA regulation. The correlations of all inter- and intra- spike-ins R^2^ ≥ 0.95 are generally accepted as a gold standard of good quality of labelling reaction, hybridization, technical variability and calibration. In this study, R^2^ for all samples were ~0.99. The dual colour labelling with Hy3^TM^ and Hy5^TM^ technique used in this study offers the advantage of Lowess normalization to minimize systematic variations. Our data showed M = 0 after normalization, therefore the difference between the two channels was dependent on the average Hy3^TM^ and Hy5^TM^ intensity level of miRNAs, and thus the accuracy was greatly increased. This array consists of approximately 3100 capture probes which are complementary to human, mouse, rat and their related sequences. Since miRNA sequences are highly conserved between species^24^, it was reasonable to expect that this array could be used to detect the conserved miRNAs from other species, e.g. sheep, with human, mouse and rat. In combination with NGS, this is a fast but reliable way to identify novel sheep miRNAs.

Of note, approximately half the miRNAs detected by NGS and a third by array overlapped. This finding is consistent with previous reports^25^. Although the techniques themselves are reliable and reproducible, systematic errors may affect results. For example, the adaptor ligation and structure might affect sequencing-based reading^26^. As a hybridization-based technique, miRNA array is highly sensitive but lacks specificity. In this study, NGS detected high counts of oar-miR-133, while array yielded high expression of hsa-/mmu-/rno-miR-133a-3p, which is one nt longer at the 5′ end compared to oar-miR-133. Substantial bias depending on base composition has recently been reported by Backes *et al*.^25^ with NGS shown to favor uracil-rich miRNAs while array favored guanine-rich miRNAs. Adding up both NGS and Array detected-miRNAs (taking the overlap into account), there were total of 1291 miRNAs. More than likely they are all true oar-miRNAs. Currently, stem-loop qPCR is the golden standard for validation. Therefore, we further selected 23 miRNAs with NGS and array intensities ranging from low to high for qPCR validation. These miRNAs were all detected by qPCR and the intensity negatively correlated to the Cq value.

MiR-1, miR-133, miR-499 and miR-208 are highly enriched myocardial miRNAs^27, 28^ and are highly conserved across multiple species including human^29^, mouse^30^ rat^31^ and porcine^32^. Oar-miR-133 is currently the only cardiac specific miRNA listed in miRBase 21. Whether the other cardiac enriched miRNAs are conserved and expressed in the sheep heart was not previously known. In this study, this panel of cardiac-specific miRNAs was reviewed by NGS and the findings reinforced by microarray and qPCR measurement. The most abundant cardiac-specific miRNA-133 in the sheep heart was oar-miR-133 which has one nt different from hsa-/mmu-/rno-miR-133a-3p (previously hsa-miRNA-133). While another group has reported that miR-133a is highly expressed in the sheep heart^17^, they did not, as has been done in the present study, specify expression of the different isoforms. Hsa-/mmu-miR-1a-3p, identical to rno-miR-1b, and hsa-/mmu-miR-208b-3p, was also highly expressed in the sheep LV. Human miRNA-1 has two isoforms, miR-1-1 and miR-1-2, which are encoded by two different genomic loci^33^. Similarly, two miRNA-1 encoded loci are found in the sheep. Two isoforms of miRNA-208-a/b have also been reported in humans, with miRNA-208a exclusively expressed in the heart and miRNA-208b found in both cardiac and skeletal muscle^34^. Our data indicate that hsa-/mmu-miR-208b-3p (previously miR-208b) was the main miR-208 isoform expressed in the sheep heart. MiR-499b is the antisense of miR-499a, and it was previously unclear whether miR-499b is expressed in the heart^20^. The current study not only confirms that both forms are highly expressed in the heart, but is the first to demonstrate the presence of this miRNA in ovine cardiac tissue.

Ischemia/reperfusion injury, heart failure, cardiac fibrosis/remodeling, etc., affect both gene and miRNA expression in cardiovascular disease. In depth understanding of their changes may lead to the discovery of novel therapeutic targets for the treatment of cardiovascular diseases^35, 36^. In the present study we further examined the regulation of miRNA in ovine HF and following recovery. Using miRNA array analysis we found 125 and 95 differentially expressed miRNAs between Cont vs. HF and Cont vs. HF-R, respectively. Validation of a selected few by qPCR identified 10 miRNAs - miR-133b-3p, miR-208b-3p, miR-21-5p, miR-125a-5p, miR-125b-5p, miR-126-3p, miR-210-3p, miR-29a-3p, miR-494-3p and miR-320a, that were significantly up-regulated in HF myocardium compared to normal controls. Most of these returned to baseline expression levels following recovery from HF. The up-regulation of these miRNAs in HF is supported by studies demonstrating that serum miR-125a-5p is significantly increased in human HF^3^, and is one of the most abundant miRNA in pericardial fluid from HF patients undergoing open-heart surgery^37^. Similarly, MiR-29a is reported to be a key regulator of cardiac fibrosis and hypertrophy^38, 39^, and miR-21 is enhanced in the fibroblasts of failing hearts^40^. MiR-210-3p is closely related to hypoxia^41, 42^, and interestingly, both miR-320a and miR-494 are reported to be associated with cardiac apoptosis induced by ischemia^43, 44^.

## Conclusions

A total of 637 miRNAs were detected in the current study. Of these, 186 were perfectly conserved with human, mouse and rat miRNAs and are unequivocally novel oar miRNAs, while a further 89 showing 70–99% alignment are also likely to be novel oar miRNAs. For the first time we report that not only are the four cardiac-enriched miR-1, miR-133, miR-499 and miR-208 highly expressed in sheep LV, but also provide information on their isomiRs. In addition, we detected 83 known oar-miRNAs on miRBase. Importantly, we demonstrated the significant up-regulation of 10 miRNAs in HF, with most returning to normal control levels following recovery from HF. This study has markedly expanded the known oar-miRNA database, thereby providing a more solid foundation with which to facilitate miRNA research in sheep models of cardiovascular disease.

## Methods

### Surgical preparation and study protocol

The study protocol was approved by the Animal Ethics Committee of the University of Otago, Christchurch, New Zealand. All methods were carefully performed in accordance with the relevant guidelines and regulations in this study. Twenty seven sheep (Coopworth ewes; 47–60 kg; Lincoln University Farm, Christchurch, New Zealand) were separated into three groups: (**i**) Normal controls (Cont; n = 13), (**ii**) Heart Failure (HF; subjected to rapid LV pacing at ~220 bpm for 13 days; n = 6) and (**iii**) HF Recovery (HF-R; sampled 26 days following cessation of pacing; n = 8).

The sheep were initially instrumented via a left lateral thoracotomy under general anesthesia (15 mg/kg thiopentone induction; 2.5% isoflurane maintenance) as previously described^45^ to allow the measurement of heart rate (HR), mean blood pressure (MBP), atrial pressure and cardiac output (CO). For HF and HF-R groups, a 7 French His-bundle electrode was implanted subepicardially in the LV wall for subsequent pacing. During the study the sheep were held in metabolic cages with free access to water and a standard laboratory diet. Prior to sacrifice, hemodynamic parameters were recorded (PowerLab Systems; ADInstruments, Dunedin, New Zealand) and blood samples taken for the measurement of atrial natriuretic peptide (ANP) and B-type natriuretic peptide (BNP)^10^. The sheep were euthanized (100 mg/kg IV Pentobarb 300) and LV tissue samples collected and snap frozen in liquid nitrogen and stored at −80 °C. Total RNA were extracted using TRIzol^®^ Reagent (Ambion, Austin, TX, USA) and overnight isopropyl precipitation according to the manufacturer’s protocol with integrity numbers (RINs) ≥6.5 (Bioanalyzer 2100, Agilent Technology, CA, USA).

### Next generation deep sequencing (NGS) to detect novel oar-miRNAs

A total of 100 ng RNA from each group of pooled Cont, HF and HF-R was converted into three small RNA libraries using a NEBNext® Ultra™ DNA Library Prep Kit for Illumina® (New England Biolabs Inc., Ipswich, MA, USA) according to the manufacturer’s instructions. Each RNA sample was ligated with adaptors to its 3′ and 5′ ends and converted into cDNA before pre-amplification with specific primers containing sample-specific indexes. After 15 cycles, pre-PCR cDNA was purified on QiaQuick columns (Qiagen, Hombrechtikon, Switzerland) and the insert efficiency evaluated by the 2100 Bioanalyzer on high sensitivity DNA chips (Agilent Inc., Waldbronn, Germany). The miRNA cDNA were size fractionated (LabChip XT, Caliper Inc., Hopkintin, MA, USA) and bands representing adaptors and 15–50 base pair (bp) inserts were excised and collected. Based on quality of the inserts and the concentration measurements the 3 libraries were assembled in equal concentrations. The libraries were finally quantified again with qPCR and sequenced on the Illumina NextSeq. 500 system (Illumina Inc., San Diago, CA, USA).

The raw NGS reads were subjected to data quality control via measurement of the quality score (Q-score, a prediction of the probability of an incorrect base-call)^21^ and filtered to remove background reads of <15 nucleotides (nts). The remaining sequences with lengths between 15 to 30 nts were analyzed. The sequence analyzing pipeline was carried out by performing alignment with known miRNAs in miRBase v21 with Blast, and followed by mapping to the sheep genome (NCBI:ftp://ftp.ncbi.nlm.nih.gov/genomes/all/GCF_000298735.2_Oar_v4.0) with Bowtie Oar v4.0. NGS sequences with at least 10 counts and homology >70% (read length over miRNA length) were included. Results of the NGS data have been deposited into GEO database under the accession number GSE87468.

### MicroRNA array to identify oar-miRNA and regulations in HF

MiRNA profiling was performed using LNA™-modified oligonucleotide (Exiqon, Denmark) probes (miRBase v19.0). Individual samples of 500 ng RNA (2 Cont, 4 HF and 3 HF-R) were labeled with Hy3™ and Hy5™ fluorescent (miRCURY LNA™ miRNA Hi-Power Labeling Kit, Hy3™/Hy5™, Exiqon, Denmark). The labeled RNA samples were mixed pair-wise and hybridized to the miRCURY LNA™ microRNA Array capture probes (7th Generation - human, mouse and rat; Exiqon, Denmark, Tecan HS4800™ hybridization station, Tecan, Austria). The slides were scanned using the Agilent G2565BA Microarray Scanner System (Agilent Technologies, Inc., USA).

Data were analyzed using ImaGene® 9 (miRCURY LNA™ microRNA Array Analysis Software, Exiqon, Denmark). Following background correction and Lowess (LOcally WEighted Scatterplot Smoothing) global normalization, the threshold for detected miRNA was 1.2 times above 25^th^ percentile of the overall signal intensity (analysis performed using R/Bioconductor). To investigate miRNA regulation in HF and HF-R, miRNA intensity was log2 transformed. Those miRNAs which exhibited absolute fold changes ≥1.2 or ≤−1.2 with a p-value < 0.05 (Student’s t-test) underwent further analysis using GeneSpring GX. Results of the microarray data have been deposited into GEO database under the accession number GSE87449.

### Stem-loop qPCR to validate miRNA regulation in HF

The qPCR experiments were followed the MIQE guidelines^46^. Total of 27 RNA samples from Cont n = 13, HF n = 6 and HF-R n = 8 were reverse transcribed into single-stranded cDNA using High Capacity cDNA Reverse Transcription (Applied Biosystems, Foster City, CA, USA) and TaqMan miRNA RT kits (Applied Biosystems). Real-time PCR was carried out using first strand cDNA with TaqMan Universal MMIX II with UNG (Applied Biosystems). Quantitative PCR parameters for cycling were as follows: 50 °C for 2 min and 95 °C for 10 min, followed by 40 cycles of 95 °C for 15 s and 60 °C for 1 min (CFX96 Real-Time System, Bio-Rad, Singapore). All reactions were performed in a 20 µl reaction volume in triplicate. U6 was used as the housekeeping reference gene. Data were analyzed using the 2^−ΔΔCT^ method^47, 48^.

### Statistical analysis

Data are expressed as Mean ± SEM unless otherwise specified. Hemodynamic and neurohumoral data from the 3 groups (Cont, HF and HF-R) were compared by independent Student’s *t*-test. NGS, miRNA array and Stem-loop qPCR data analysis are described in their specific sections. In general, differences between groups were assessed by one-way analysis of variance (ANOVA) followed by Bonferroni post-hoc analysis or unpaired two tail t-tests as appropriate (Graph Pad Prism, San Diego, CA, USA). A p value of <0.05 was considered statistically significant.

### Availability of data and materials

The microarray and NGS data have been deposited into GEO database under the accession numbers GSE87449 (https://www.ncbi.nlm.nih.gov/geo/query/acc.cgi?acc=GSE87449) and GSE87468 (https://www.ncbi.nlm.nih.gov/geo/query/acc.cgi?acc=GSE87468) respectively.

## Electronic supplementary material

Table S1

Table S2

Table S3

Table S4

Table S5

Table S6
